# *CsSPX3-CsPHL7-CsGS1/CsTS1* module mediated Pi-regulated negatively theanine biosynthesis in tea (*Camellia sinensis*)

**DOI:** 10.1093/hr/uhae242

**Published:** 2024-08-30

**Authors:** Zhouzhuoer Chen, Zhixun Yu, TingTing Liu, Xinzhuan Yao, Shiyu Zhang, Yilan Hu, Mingyuan Luo, Yue Wan, Litang Lu

**Affiliations:** The Key Laboratory of Plant Resources Conservation and Germplasm Innovation in Mountainous Region (Ministry of Education), College of Life Science, Guizhou University, Guiyang 550025, China; The Key Laboratory of Plant Resources Conservation and Germplasm Innovation in Mountainous Region (Ministry of Education), College of Life Science, Guizhou University, Guiyang 550025, China; The Key Laboratory of Plant Resources Conservation and Germplasm Innovation in Mountainous Region (Ministry of Education), College of Life Science, Guizhou University, Guiyang 550025, China; College of Tea Science, Institute of Plant Health & Medicine, Guizhou University, Guiyang 550025, China; The Key Laboratory of Plant Resources Conservation and Germplasm Innovation in Mountainous Region (Ministry of Education), College of Life Science, Guizhou University, Guiyang 550025, China; The Key Laboratory of Plant Resources Conservation and Germplasm Innovation in Mountainous Region (Ministry of Education), College of Life Science, Guizhou University, Guiyang 550025, China; The Key Laboratory of Plant Resources Conservation and Germplasm Innovation in Mountainous Region (Ministry of Education), College of Life Science, Guizhou University, Guiyang 550025, China; Huaneng Clean Energy Research Institute, Beijing 102209, China; The Key Laboratory of Plant Resources Conservation and Germplasm Innovation in Mountainous Region (Ministry of Education), College of Life Science, Guizhou University, Guiyang 550025, China

## Abstract

Phosphorus (P) is the macronutrients essential for the development and growth of plants, but how external inorganic phosphate (Pi) level and signaling affect tea plant growth and characteristic secondary metabolite biosynthesis are not understood. Theanine is major secondary metabolites, and its contents largely determine tea favor and nutrition qualities. Here, we found theanine contents in tea leaves and roots declined as Pi concentration increased in tea plants after Pi feeding. The transcriptome analysis of global gene expression in tea leaves under Pi feeding suggested a wide range of genes involved in Pi/N transport and responses were altered. Among them, *CsSPX3* and *CsPHL7* transcript levels in response to Pi feeding to tea plants, their expression patterns were generally opposite to these of major theanine biosynthesis genes, indicating possible regulatory correlations. Biochemical analyses showed that CsSPX3 interacted with CsPHL7, and *CsPHL7* negatively regulated theanine biosynthesis genes *CsGS1* and *CsTS1*. Meanwhile, VIGS and transient overexpression systems in tea plants verified the functions of *CsSPX3* and *CsPHL7* in mediating Pi-feeding-repressed theanine biosynthesis. This study offers fresh insights into the regulatory mechanism underlying Pi repression of theanine biosynthesis, and the *CsSPX3-CsPHL7-CsGS1/CsTS1* module plays a role in high Pi inhibition of theanine production in tea leaves. It has an instructional significance for guiding the high-quality tea production in tea garden fertilization.

## Introduction

Tea plants (*Camellia sinensis* (L.) O. Kuntze) are valuable crops with its leaves being processed into various popular teas. As the second largest drink in the world, following water, teas have distinct flavors and many health benefits [1]. One of the distinctive secondary metabolites that gives teas their distinct flavors and health benefits is theanine [2]. As a non-protein amino acid secondary metabolite in significant levels in tea roots and young leaves, theanine has unique pharmacological and chemical properties to not only improve human health, but also directly affect tea sensory qualities, and participate in the metabolism and physiological processes in the tea tree [3]. The biosynthesis of theanine has been studied extensively, and several key enzymes, including CsTS1, CsGS1a, CsGDH, and CsAlaDC, are shown to participate in theanine biosynthesis pathway [4, 5]. Many environmental variables, including light and temperature, or cultivation conditions, such as nitrogen (N) and phosphorus (P) fertilizers, have an impact on the expression of the genes encoding these essential synthesis enzymes and the theanine concentrations in tea leaves [6].

As the most abundant constrictive macroelements in plants P and N availability have significant impacts on plant growth and development and the production of theanine, caffeine, and tea polyphenols [7]. While the effects of N on tea plant vegetation and theanine contents have been well studied in tea plants [8], phosphate (Pi) effects on tea plants are poorly understood. In 2012, Lin et al showed that the contents of total polyphenols, total free amino acids and theanine decreased in Pi-deficient ‘Huangguanyin’ [9], but recently a study indicated that theanine decreased in ‘Fengqing’ but increased in ‘Longjing 43’, whereas catechins increased in ‘Fengqing’ but decreased in ‘Longjing 43’ under Pi deficiency, the effects of Pi deficiency or Pi excess on the secondary metabolism of theanine and catechins in tea roots and buds are complex, with variety dependence and concentration dependence [10, 11]. A previously study elucidated how Pi/N application affects the accumulation of quality-related compounds and the mechanism of nutrients affecting tea metabolism [12], and the contents of glutamic acid and theanine showed a negative correlation with Pi nutrient content. Due to the extremely low concentration of soluble Pi in soil, plants have developed an extensive array of methods to deal with Pi deficiency [7].

The SPX protein family contains the SPX domain, called after SYG1, PHO81, and Xpr1, have been shown as important Pi sensing (such as *Arabidopsis* SPX1, 2, 3, 4), transport (such as PHO1), and regulation (such as PHO2 E3 ubiquitin ligase) functions in plants to regulate Pi homeostasis [13]. In plants, based on various domains in their structures, the SPX family is divided into four subfamilies: SPX, SPX-EXS, SPX-MFS, and SPX-RING [14]. AtSPX1-3 proteins are critical for sensing internal and external Pi levels for adversely affecting plant Pi homeostasis [15, 16]. *AtSPX1* and *AtSPX3* were strongly induced to express with unique dynamic patterns by phosphate starvation, but *AtSPX2* was slightly induced and *AtSPX4* was inhibited [14]. *PHR1* and its homolog *PHL1* are regarded as crucial elements of the central regulatory system that control the transcriptional response to Pi starvation in plants [17]. The transcriptional activity of *AtPHR1/OsPHR2* is inhibited by *AtSPX1* and *OsSPX1*, respectively [15, 18]. Rice *SPX1*, *SPX2*, *SPX3*, and *SPX5* accumulate in shoots and roots under Pi starvation [18, 19]. The nucleus-localized SPX1 and SPX2 proteins in *Arabidopsis* interact with the GARP transcription factor *AtPHR1* to induce *PHR1* degradation and regulates Pi starvation-induced gene expression along with its homologs PHR1-like (PHL1) [20]. *PHL1* is a nearby homolog of *PHR1*, When *PHR1* and *PHL1* were simultaneously knocked out, about 70% of the low-Pi response gene expression was affected [21]. *PHL2*, *PHL3*, and *PHL4* also regulates the low Pi transcriptional response [22]. *PHL2* and *PHL3* specifically attach to a DNA sequence within the promoter of the Pi starvation-induced acid phosphatase *AtPAP10*, leading to the activation of its transcription [23]. Pi starvation increased the transcription and accumulation of proteins for PHL2 and PHL3, in turn, a majority of the Pi starvation-induced genes are down-regulated in phl2 mutants, as revealed by RNA-sequencing analysis, suggesting that *PHL2* is an essential component of the central regulatory system as well [24]. *PHR1* and *PHL* regulation of low-Pi responses is N status-dependent and closely related to *HRS1/NIGT1* and N-status changes induced N responses and signaling and N assimilation, such as *NLP*, *NRT1* and *NRT2*, *GS*, and *GDH* [25]. Therefore, N and Pi nutrient interaction and signaling are extensively studied, including SPX interaction with HRS1 to regulate N assimilation in rice [26]. However, the role of Pi fertilization, and Pi signaling, including SPX and PHL, in regulating theanine biosynthesis in tea leaves remains largely unknown.

In the investigation, we found the theanine content in tea leaves and roots gradually reduced by Pi feeding and increasing Pi concentration in tea leaves. Transcriptome data analysis showed that Pi feeding differentially regulated *SPX*, *PHR*, and *PHL* genes. However, the expression levels of Pi signaling factors *CsSPX3* and *CsPHL7* were up-regulated, in an opposite trend to theanine content. Molecular biochemical studies revealed the physical interaction between CsSPX3 and CsPHL7, regardless of Pi concentration. CsSPX3 played an additive role in the negative regulation of *CsTS1* and *CsGS1* by CsPHL7. CsSPX3 synergized with CsPHL7 activity in the repression of the expression of *CsGS1* and *CsTS1*, thereby reducing theanine accumulation. Knockdown of *CsSPX3* and *CsPHL7* in tea plants by using virus-induced gene silencing (VIGS) resulted in increased theanine contents, whereas the inhibited theanine accumulation in tea plant leaves was observed upon overexpression of Cs*SPX3* and Cs*PHL7* through the Agrobacterium-mediated transient transformation. The study reveals the *CsSPX3-CsPHL7-CsGS1/CsTS1* module may mediate Pi regulation of theanine biosynthesis, which will facilitate tea plant breeding or cultivation to enhance the theanine content.

## Results

### The changes of theanine in tea plants treated with different concentrations of phosphorus and the detection of phosphorus content

**Figure 1:**
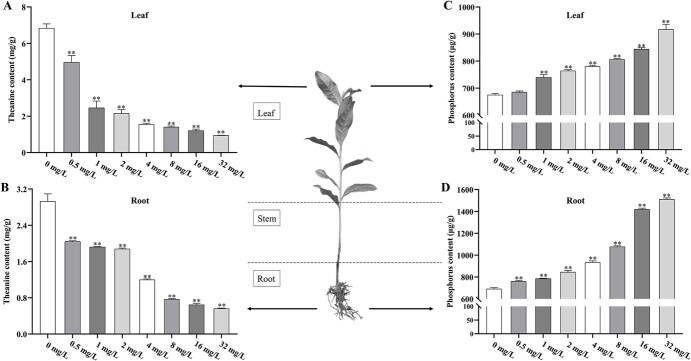
The changes of theanine and phosphorus content in tea plants treated with different concentrations of phosphorus. **(A)** Theanine content in tea leaves on the 20th day of different phosphorus concentration treatment. **(B)** Theanine content in tea roots on the 20th day of different phosphorus concentration treatment. **(C)** Phosphorus content in tea leaves on the 20th day under different phosphorus concentration treatments. **(D)** Phosphorus content in tea roots on the 20th day under different phosphorus concentration treatments. The error bars represent the mean ± SE of the three independent experiments. ^*^ indicate significant differences at *p* < 0.05 by Duncan’s multiple range tests. SE, standard error.

We first examined theanine content in both tea leaves and roots treated with different phosphorus concentrations (0 mg·L^−1^, 0.5 mg·L^−1^, 1 mg·L^−1^, 2 mg·L^−1^, 4 mg·L^−1^, 8 mg·L^−1^, 16 mg·L^−1^, 32 mg·L^−1^), and found that theanine content decreased significantly with the increasing Pi treatment concentration (Figure 1A, B). In addition, the amount of phosphorus in leaves and roots under different phosphorus treatments was detected. It showed an opposite trend with theanine content: the Pi contents in leaves and roots increased (Figure 1C, D), but theanine contents decreased over the increasing phosphorus treatment concentrations. In addition, we also detected catechins and caffeine contents in leaves and roots treated with different phosphorus concentrations and hypothesized that Pi also impacts catechins and caffeine synthesis. Indeed, similar to those reported in other plants, the tea plants grown in the low Pi hydroponic nutrient solution produced more catechins and caffeine (Figure S1).

### Transcriptome revealed Pi/N transport, sensing, responsive and N assimilation gene expression changes by phosphorus treatment

**Figure 2:**
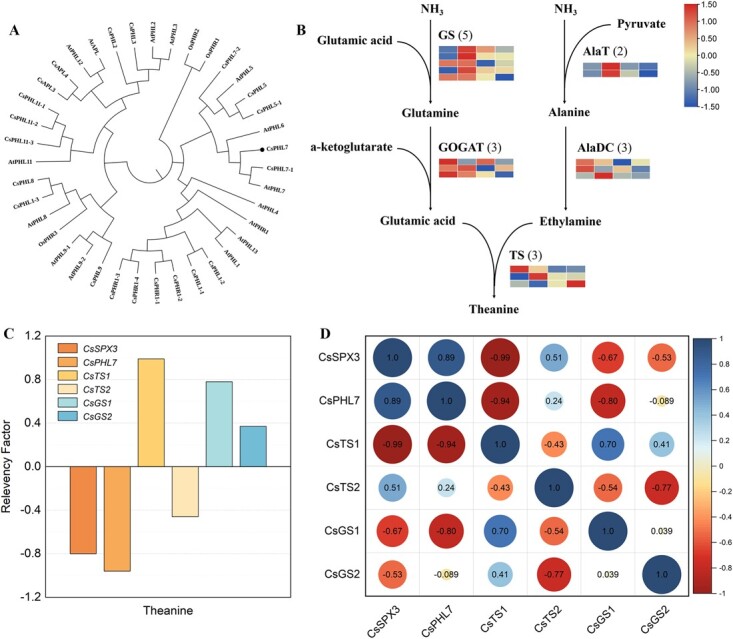
Correlation analysis between gene expression levels and theanine. **(A)** Phylogenetic analysis of PHR/PHL in various species. **(B)** DEGs heat map of theanine biosynthesis pathway and related genes. The number in brackets after each gene name represents the number of corresponding DEGs. **(C)** Correlation analysis of the expression of candidate genes and the contents of theanine. **(D)** Pearson analysis on the expression levels. The size of circles indicated the degree of correlation. Blue means positive correlation, and red means negative correlation.

We performed transcriptome analysis on the leaves under Pi treatment at different concentrations to gain a deeper understanding of the molecular mechanisms underlying the contrary trends of theanine with Pi levels in tea plants. Through WGCNA analysis, all genes were divided into 15 modules, showing the correlation analysis between the changes of theanine content in tea leaves treated with different concentrations of phosphorus and the differential genes of modules. The cyan module was significantly negative correlated with theanine (R > 0.7, *p* < 0.01), and the expression network was constructed based on the genes of the cyan module (Figure S2). On the basis of a phylogenetic tree analysis, we obtained PHR/PHLs related to Pi signal response in *C. sinensis* with their homologs in *Arabidopsis* and rice (Figure 2A). In addition, the phylogenetic analysis of all SPX domain proteins identified seven SPX family members from *C. sinensis* (Figure S3). Among all the DEG genes, we screened *CsSPX3* which containing SPX domain and a phosphorus-starvation response transcription factor *CsPHL7* significantly respond to Pi signal. Moreover, the related genes on theanine synthesis pathway and the theanine transporter gene *CsAPP* also showed changes in expression levels in response to varying phosphorus treatments. The majority of the genes, including *CsTS* and *CsGS*, showed down-regulated expression, suggesting that the addition of phosphorus inhibited their expression (Figure 2B). Correlation analysis showed that *CsSPX3* and *CsPHL7* showed a strong association between theanine and the expression of theanine synthetase 1 (TS1) and glutamine synthetase GS1 (Figure 2C,D). The majority of *CsAPPs* had down-regulated expression, with the exception of *CsAPP7* (Figure S2D).

**Figure 3:**
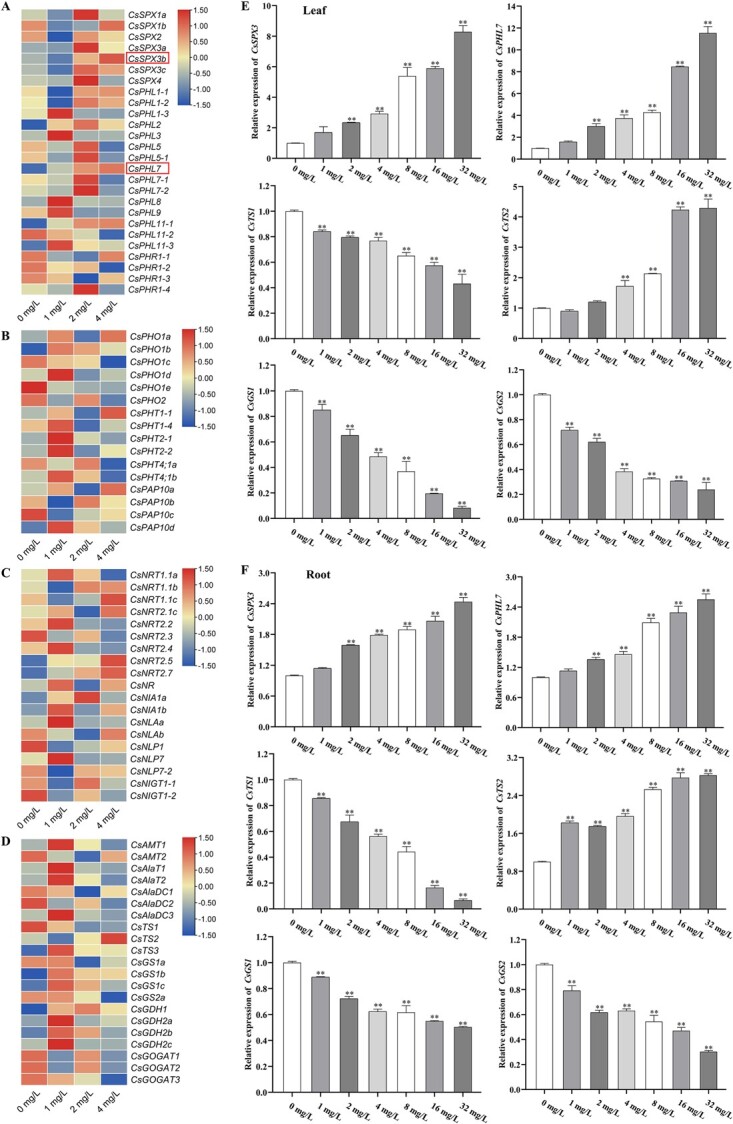
Transcriptome profile of phosphorus-treated RNA-seq and differential expression of related genes. **(A)** RNA-seq transcriptome profiles of SPX and PHL genes under different phosphorus concentrations. **(B)** RNA-seq transcriptome profiles of phosphorus transport genes under different phosphorus concentrations. **(C)** RNA-seq transcriptome profiles of nitrogen transport genes under different phosphorus concentrations. **(D)** RNA-seq transcriptome profiles of theanine synthesis-related genes under different phosphorus concentrations. **(E)** The relative level of expression of *CsSPX3*, *CsPHL7*, *CsTS1*, *CsTS2*, *CsGS1,* and *CsGS2* in tea leaves*.***(F)** The relative level of expression of *CsSPX3*, *CsPHL7*, *CsTS1*, *CsTS2*, *CsGS1,* and *CsGS2* in tea roots*.* The error bars represent the mean ± SE of the three independent experiments. ^*^ indicate significant differences at *p* < 0.05 by Duncan's multiple range tests. SE, standard error.

Transcriptome data showed that most Pi sensing and signaling genes, including many *PHT*, *PAP*, *SPX*, *PHR*, *PHL*, *PHO1*, and *PHO2* homolog genes in tea plants, were changed by Pi feeding (Figure 3A, B), Moreover, N sensing, responsive, assimilation, and signaling genes, such as *NRT1;1*, *NRT2*, *NR*, *NIA*, *GS*, *GDH*, *GOGAT*, *NLAs*, *NLP* homolog genes were also changed drastically in their expression levels (Figure 3C,D), indicating the interactions between Pi and N nutrition and signaling, as observed in most model plants. *AtNIGT1* is induced by nitrate and Pi starvation dependent of *AtNRT1.1*, but repressed by Pi supply and AtNIGT1 protein accumulation reduced by phosphate starvation [27]. Additionally, *NIGT1* represses the phosphate starvation response's repressor genes, such as *AtSPX1/2/4* and *AtPHO2*, which activates phosphate utilization [28]. *AtNLP7* and *AtPHR1* regulate the N and Pi starvation-induced expression of *AtNIGT1s*, which integrates *AtNIGT1s* into the primary N/Pi signaling pathway [29]. Homologs (CsNIGT1-1 and CsNIGT1) of AtNIGT1, a critical protein integrating both N and Pi signaling, were also down- or up-regulated by Pi feeding (Figure 3C). At the same time, transcriptome data showed that the phosphate transporter gene *CsPHT* and *CsPHO* showed down-or up-regulated by Pi supply, but most of them were down-regulated (Figure 3B).

Further analysis by qRT-PCR showed that phosphorus treatment increased *CsSPX3* and *CsPHL7* expression levels in leaves and roots (Figure 3E, F). Under conditions of high phosphorus concentration, the relative expression levels of *CsSPX3* and *CsPHL7* rose, while under conditions of low phosphorus, the opposite was true. Furthermore, there was a down-regulation of *CsTS1*, *CsGS1*, and *CsGS2* expression levels in roots and leaves (Figure 3E, F). These data suggest that the *CsSPX3* and *CsPHL7* expression levels were negatively correlated with theanine accumulation in tea leaves and roots.

### SPX3 interacted with PHL7 independent of Pi concentration

**Figure 4:**
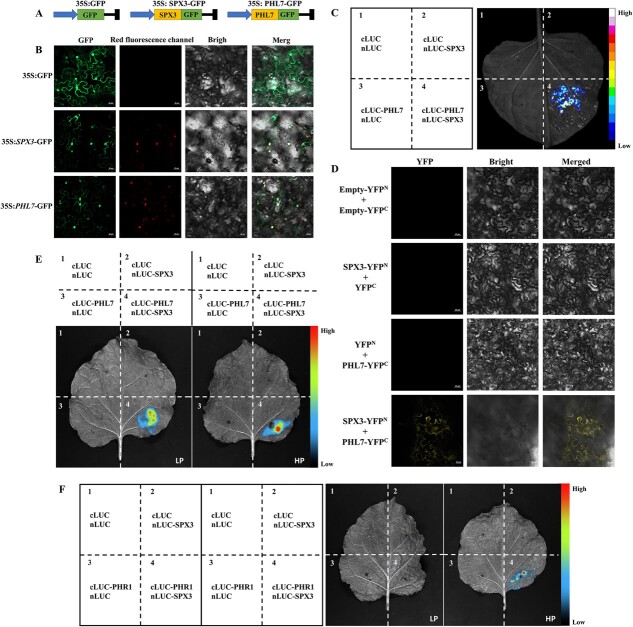
The SPX3-PHL7 interaction is not significantly Pi-dependent but the SPX3-PHR1 interaction is Pi-dependent. **(A)** Schematic of the vectors used for SPX3 and PHL7 subcellular localization. **(B)** Subcellular localization of SPX3 and PHL7 in tobacco leaves. Scale bar = 20 μm. **(C)** Luciferase complementation assay (LCI) of SPX3 and PHL7 interactions in *N. benthamiana* leaves. **(D)** BiFC assays for determination of SPX3 and PHL7 interactions in *N. benthamiana* leaf cells. Scale bar = 20 μm. **(E)** The interaction between SPX3 and PHL7 under HP and LP conditions. **(F)** The interaction between SPX3 and PHR1 under HP and LP conditions.

Firstly, we investigated the subcellular localization of SPX3 and PHL7. We observed the green fluorescent protein (GFP) fusion expression vectors (35S: SPX3-GFP and 35S: PHL7-GFP) were used to express SPX3 and PHL7, respectively, while the empty plasmid (35S: GFP) served as a control. The findings demonstrated that GFP signal was detected in the entire cells expressing 35S: GFP control, while both fusion proteins, 35S: SPX3-GFP and 35S: PHL7-GFP localized to the nucleus as well as the cell membrane (Figure 4B). And Pi levels had no effect on the subcellular location (Figure S4).

To explore whether there is interaction between SPX3 and PHL7 in plant cells, the coding sequences of SPX3 and PHL7 were fused to the N- and C-terminus of luciferase (SPX3-nLUC and PHL7-cLUC), and they were transiently co-transformed into *N. benthamiana* leaves and treated with 1 mM fluorescein potassium salt. The results indicated that the combination of SPX3-nLUC+PHL7-cLUC produced a luciferase signal while the negative control combination (PHL7-cLUC+nLUC; cLUC+SPX3-nLUC; cLUC+nLUC) did not, confirming the interaction between SPX3 and PHL7 (Figure 4C). Then we carried out BiFC to further verify the SPX3-PHL7 interaction in tobacco leaves. It demonstrated that tobacco cells co-transformed with SPX3 and PHL7 produced yellow fluorescence in their cell membranes, while no fluorescence was seen in the corresponding negative control (SPX3-YFP^N^+YFP^C^; YFP^N^+PHL7-YFP^C^) (Figure 4D).

To determine if the SPX3-PHL7 interaction is Pi-dependent in vivo, we conducted LUC studies in tobacco seedlings grown under high Pi(HP;200 μM Pi) and low Pi(LP;5 μM Pi) conditions respectively. It is well established that inositol polyphosphates (InsPs) facilitate the binding of the SPX protein to the targets of transcription factors (such PHRs) [30, 31]. We tested the SPX3-PHL7 interaction under various InsP6 concentrations and discovered that the combination of SPX3-nLUC and cLUC-PHL7 could notice significant fluorescence signals no matter the high or low concentration of InsP6 was added (Figure 4E). But in rice and *Arabidopsis*, SPX proteins interact with PHR to suppress its function under high Pi circumstances [15]. We found *C. sinensis* contain six PHR-like proteins to use phylogenetic analysis (Figure S5). Therefore, we also verified the Pi dependence of SPX3 interaction with *C. sinensis* PHR homologs PHR1. The findings indicated that SPX3 interacted with PHR1 in high Pi levels, while no interaction was observed in low Pi levels. (Figure 4F).

### SPX3-PHL7 interaction represses *TS1* and *GS1* expression and reduces theanine accumulation in tea plants

**Figure 5:**
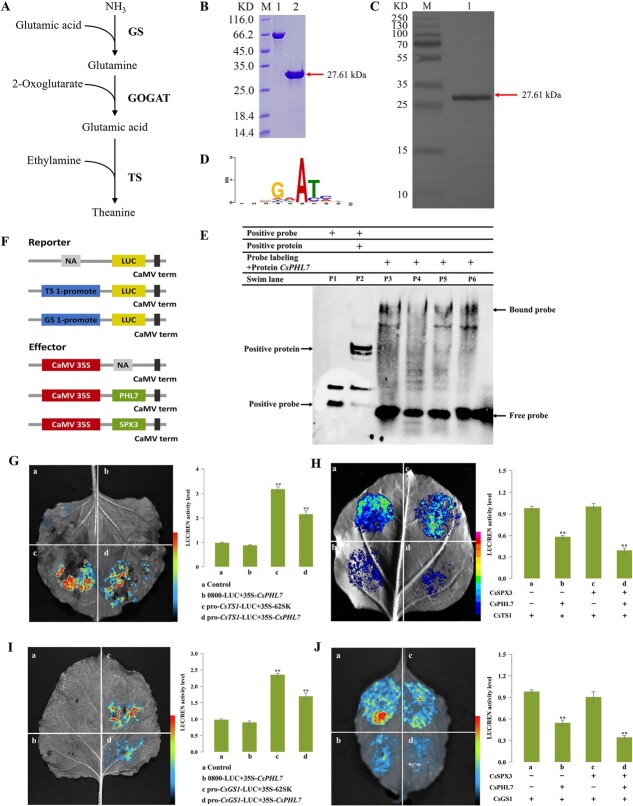
CsPHL7 bond to the *CsTS1* and *CsGS1* promoters and repressed the transcription of *CsTSI* and *CsGS1*. **(A)** The biosynthesis pathway of theanine in tea plants. **(B)** SDS polyacrylamidegel electrophoresis detected the protein of PHL7-His were successfully extracted. M: Quality standard for protein molecules; 1: 0.5 mg/mL BSA; 2: Purified samples. **(C)** Protein Western Blot identification analysis. M: Quality standard for protein molecules; 1: Purified sample. Red arrow denotes target protein bands with corresponding molecular weight. **(D)** The predicted binding sequence motif of PHL7. **(E)** Purified recombinant protein His-PHL7 binds to the *CsTS1* promoter in EMSA assay. **(F)** Vector schematic diagram of dual-LUC reporter system. **(G)** Dual-LUC transient expression of *CsPHL7* inhibits the expression of *CsTS1*. **(H)** Dual-LUC assay in *N.benthamiana* leaves showing the effects on transcription of *CsTS1* by *CsSPX3* and *CsPHL7*. **(I)** Dual-LUC transient expression of *CsPHL7* inhibits the expression of *CsGS1*. **(J)** Dual-LUC assay in *N.benthamiana* leaves showing the effects on transcription of *CsGS1* by *CsSPX3* and *CsPHL7*. Left, representative photographs; Right, detected LUC/Renilla Luciferase (REN) activity.

The association between the PHL7 protein and the *TS1* promoter was verified by the electrophoretic mobility shift assay (EMSA). Following incubation of the purified PHL7 protein (Figure 5B, C, S6) with various DNA segments of the biotin-labeled *TS1* promoter, gel migration assays were carried out. The lane band of protein+nucleic acid considerably lags behind other lane after the PHL7 protein attaches to nucleic acid fragments of varying lengths. The PHL7 protein can bind to the *TS1* promoter sequence, as demonstrated by the gel electrophoresis findings of probes of various lengths interacting with proteins in lanes 3 to 6 (Figure 5E). The outcomes demonstrated that PHL7 and *TS1* promoters had specific binding.

The dual-LUC was employed to explore the regulating mechanism of *CsPHL7* on *CsTS1*. The tobacco leaves co-transfected with *CsPHL7* and *CsTS1* promoters produced luciferase signals, but the negative control (0800-LUC-*TS1*+62-SK) combination produced stronger luciferase signals, and its fluorescence value was substantially less than the negative control group (Figure 5G). This finding indicates that *CsPHL7* can bind and inhibit *CsTS1*. Moreover, in view of the coexistence of *CsSPX3*, *CsPHL7*, and *CsTS1* promoters, when *CsPHL7* was co-expressed with the *CsTS1* promoter, the LUC/REN ratio was lower than that of the control group. When *CsSPX3* was added, the LUC/REN ratio decreased significantly (Figure 5H). The co-expression of *CsSPX3* and *CsPHL7* inhibited the luciferase reporter gene driven by the *CsTS1* promoter. The results showed that co-expression of *CsSPX3* increased the ability of *CsPHL7* to inhibit *CsTS1* expression.

In addition, *CsTS1* exhibits a high degree of sequence similarity with *CsGSs* [32]. *CsTS1* is primarily expressed in roots and is in charge of catalyzing theanine synthesis, while *CsGS1* and *CsGS2* mostly catalyze theanine synthesis in tea leaves [33–35]. We also examined whether Cs*PHL7* binds to and regulates the *CsGS1* promoter that is highly expressed in leaves. The findings showed that *CsPHL7* could bind to and inhibit *CsGS1* promoter (Figure 5I). When *CsSPX3* was co-expressed, Cs*SPX3* could further promote Cs*PHL7* to inhibit Cs*GS1* promoter activity (Figure 5J). Meanwhile, EMSA proved that *CsPHL7* and *CsGS1* promoters also had specific binding (Figure S7).

### Silencing of *CsSPX3* and *CsPHL7* promoted theanine biosynthesis

**Figure 6:**
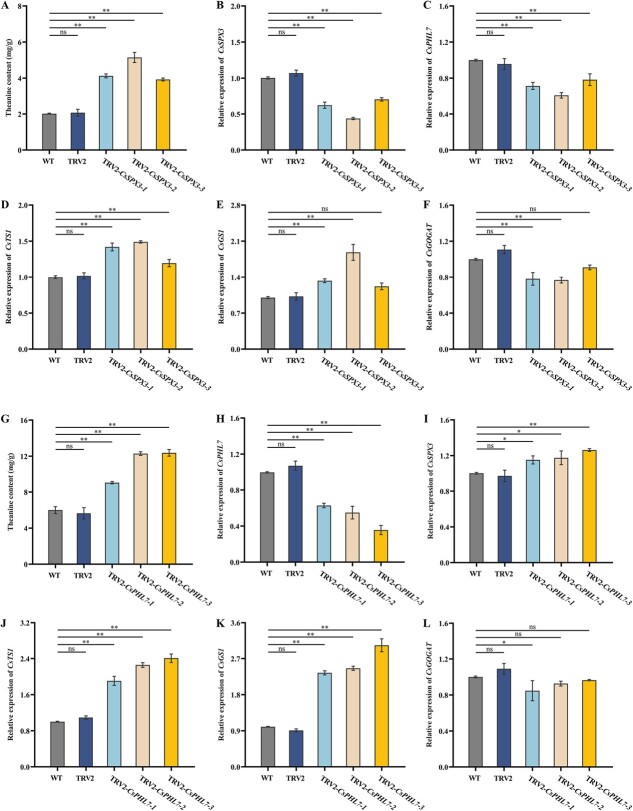
Silencing of *CsSPX3* and *CsPHL7* in tea plant leaves using VIGS. **(A)** The changes of theanine contents in WT (untreated tea cuttings), TRV2 (tea cuttings infected by TRV1+TRV2 Agrobacterium), and TRV2-*CsSPX3* (tea cuttings infected by TRV1+ TRV2-*CsSPX3* Agrobacterium). The relative expression levels of **(B)***CsSPX3*, **(C)***CsPHL7*, **(D)***CsTS1*, **(E)***CsGS1,* and **(F)***CsGOGAT* in *CsSPX3* silenced plants**.** WT, TRV2 and TRV2-*CsSPX3* are the same as above. **(G)** The changes of theanine contents in WT (untreated tea cuttings), TRV2 (tea cuttings infected by TRV1+TRV2 Agrobacterium), and TRV2-*CsPHL7* (tea cuttings infected by TRV1+ TRV2-*CsPHL7* Agrobacterium). The relative expression levels of **(H)***CsPHL7*, **(I)***CsSPX3*, **(J)***CsTS1*, **(K)***CsGS1* and **(L)***CsGOGAT* in *CsPHL7* silenced plants**.** WT, TRV2 and TRV2-*CsPHL7* are the same as above. The error bars represent the mean ± SE of the three biological replicates experiments. ^*^ indicate significant differences at *p* < 0.05 by multiple range tests. SE, standard error; TRV, tobacco rattle virus; VIGS, virus-induced gene signaling. ^*^p < 0.05 ,^**^ *p* < 0.01.

In order to further investigate whether Pi signaling components *CsSPX3* and *CsPHL7* could suppressed theanine production in tea plants, we silenced *CsSPX3* and *CsPHL7* in ‘Fuding Dabaicha’ using VIGS to test its function in theanine biosynthesis. After 40 days of injecting TRV2-*CsSPX3*-carrying *Agrobacterium tumefaciens* into tea plants, the leaves of silenced *CsSPX3* (TRV2-*CsSPX3*) and control (WT and TRV2) tea plants were harvested. qRT-PCR analysis showed that *CsSPX3* was effectively silenced in VIGS-treated tea plants, by 53%, 39%, and 63% lower than the control plants, respectively (Figure 6B). The expression level of *Cs*P*HL7* also showed a decrease (Figure 6C). Correspondingly, the expression levels of *CsTS1* and *CsGS1* were significantly increased in *CsSPX3*-silenced plant leaves (Figure 6D-F). In addition, repeated measurements of theanine contents in VIGS-mediated *CsSPX3* silencing tea plants in comparison to the control plants (TRV1+TRV2) showed that generally theanine contents increased by 1.89 times (TRV-*CsSPX3*-1), 2.36 times (TRV-*CsSPX3*-2), and 1.80 times (TRV-*CsSPX3*-3) respectively (Figure 6A).

**Figure 7:**
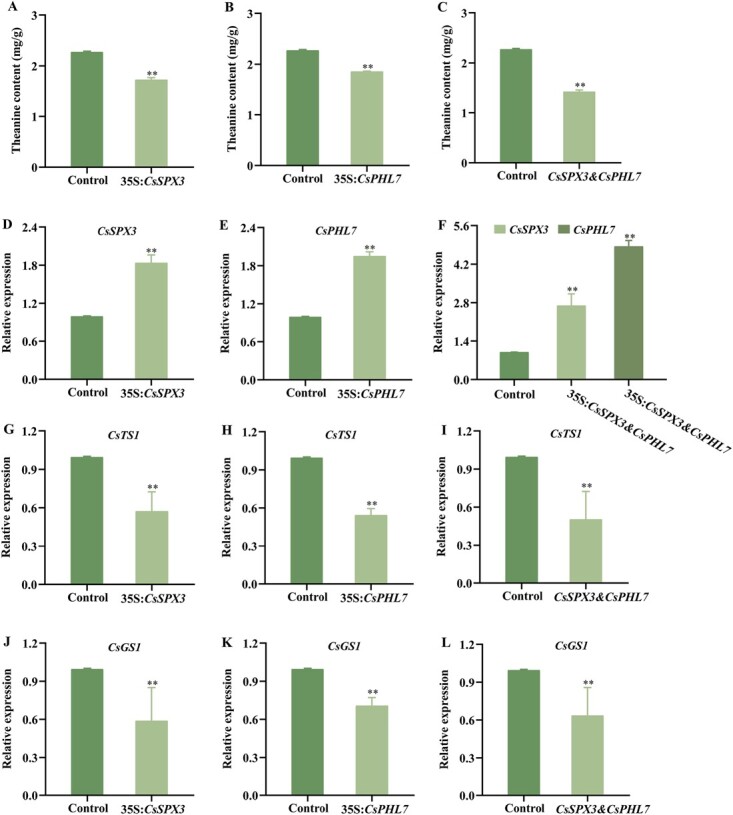
Transient overexpression of *CsSPX3* or *CsPHL7* alone and co-expression of *CsSPX3* and *CsPHL7* decreased theanine content in tea leaves. Theanine content after overexpression of **(A)***CsSPX3*, **(B)***CsPHL7* alone and **(C)** co-expression of *CsSPX3* and *CsPHL7* together. qRT-PCR analysis of **(D)***CsSPX3*, **(G)** *CsTS1* and **(J)***CsGS1* in the leaves in overexpression of *CsSPX3* lines. qRT-PCR analysis of **(E)***CsPHL7*, **(H)** *CsTS1* and **(K)***CsGS1* in the leaves in overexpression of *CsPHL7* lines.. qRT-PCR analysis of **(F)***CsSPX3* and *CsPHL7*, **(I)** *CsTS1* and **(L)***CsGS1* in the leaves in co-expression of *CsSPX3* and *CsPHL7* lines. The error bars represent the mean ± SE of the three biological replicates experiments. ^*^ indicate significant differences at *p* < 0.05 by multiple range tests. SE, standard error.

Simultaneously, the leaves of silent *CsPHL7* (TRV2-*CsPHL7*) and control (WT, TRV2) tea plants were harvested after 40 days of injection with *Agrobacterium tumefaciens* carrying TRV2-*CsPHL7*, qRT-PCR analysis showed that *CsPHL7* was successfully silenced in tea plants, and its expression levels were reduced to 55%, 49%, and 45% respectively (Figure 6H), while the expression level of *CsSPX3* slightly increased or unchanged (Figure 6I). When *CsPHL7* was silenced in tea leaves, the inhibitory effect of *CsPHL7* on *CsTS1* and *CsGS1* was relieved, both the expression level of *CsTS1/CsGS1* and the content of theanine were notably increased (Figure 6J-L), theanine content increased significantly by 1.58 times (TRV-*CsPHL7*-1), 2.10 times (TRV-*CsPHL7*-2), and 2.17 times (TRV-*CsPHL7*-3) respectively (Figure 6G). Confirming that *CsPHL7* downregulated the transcription of *CsTS1/CsGS1* to inhibit the accumulation of theanine biosynthesis. In addition, we found that compared with the control group, Pi feeding inhibited the increase of theanine content in *CsPHL7* silenced tea plants, and the silencing of *CsSPX3* also showed a similar change rule (Figure S8).

### Transient overexpression of *CsSPX3* and *CsPHL7* in tea plants repressed theanine biosynthesis

To obtain an gain-of-function tea plant mutants of *CsSPX3* and *CsPHL7* for more genetic evidence for their functions in regulating theanine biosynthesis, the suspension mixtures of *Agrobacterium tumefaciens* harboring *CsSPX3*, *CsPHL7*, or their combinations (*CsSPX3* plus *CsPHL7)* were activated and infiltrated into the second leaf of ‘Wuniuzao’, respectively, with the *Agrobacterium* harboring empty vector as controls (Figure S8). Gene expression and theanine content were detected 72 hours after the infiltration. The findings demonstrated that in contrast to the control group, the gene expression level in the tea leaves with instantaneous overexpression of *CsSPX3* and *CsPHL7* was significantly up-regulated, and the relative gene expression level of *CsSPX3* and *CsPHL7* in the *CsSPX3* and *CsPHL7* co-transfer group was higher (Figure 7D–F). Moreover, the expression level of *CsTS1* and *CsGS1* in tea leaves of *CsSPX3* and *CsPHL7* co-expression lines were higher than those in the leaves of the *CsSPX3* and *CsPHL7* lines alone (Figure 7G-L). It is vital to notice that in contrast to the control, theanine content decreased by 23%, 19%, and 37%, respectively (Figure 7A–C). The results showed that *CsSPX3* cooperated with *CsPHL7* to negatively regulate theanine biosynthesis. In addition, it was found that compared with the control group, the Pi feeding-inhibited theanine content was significantly lower in the tea leaves overexpressing *CsPHL7* or *CsSPX3* (Figure S10).

## Discussion

### Reduced theanine content in tea plants under Pi supply

Theanine is a significant secondary metabolite found in tea plant leaves, gives green tea its distinct umami and fresh flavors as well as health benefits for human neurons. Theanine thus has drawn extensive attentions of tea plant biologists, tea breeders and researchers in tea health functions [36]. Theanine biosynthesis in plants is strictly regulated by genetic, physiological, and environmental variables, such as developmental stage, hormones, temperature, light, nutrients, and various stresses [36, 37]. The effects of N fertilizers on theanine have been extensively studied, these of Pi fertilizers are less reported [11, 12, 38, 39]. As N, Pi are important to plants including tea trees, irrational phosphate fertilization can reduce the sensory quality of tea and induce the accumulation of some anthocyanins [40], it is of great interest to understand how Pi affect tea plant secondary metabolite accumulation in tea roots and leaves. Our study here examined the Pi content and theanine content in leaves and roots of tea plants under various Pi conditions. It was found that with the increase of Pi concentrations, the Pi level in tea leaves and roots increased, but the theanine content decreased significantly. We further explored the molecular mechanism for high Pi supply inhibition of theanine biosynthesis in tea leaves and roots.

### CsSPX3–CsPHL7 module mediated Pi-regulated theanine biosynthesis in tea plants

The prior study has unequivocally shown that SPX proteins play a critical function in Pi signaling network by sensing Pi homeostasis [41]. Pi- or PI6-induced *SPX* genes, then subsequently influenced the transcription of downstream genes induced by Pi starvation by negatively modulating PHR activity. This may occur through the regulation of PHR relocation from the cytoplasm to the nucleus and the reduction of PHR binding to the P1BS cis-element [13]. In rice, *OsPHR2* activity is regulated by *OsSPX1* and *OsSPX2* in a Pi-dependent way [18]. Other SPX family members, such as *OsSPX4*, are located to the cytosol and regulate the cytoplasmic-to-nuclear shuttling of *OsPHR2* in a Pi-dependent way [42]. Concurrently, a growing amount of evidence suggests that SPX proteins regulate other important plant processes, such as the transmission of nitrate signals to initiate the Pi starvation response [26, 43], disease resistance [44], iron deficiency response [45], hypoxia response [46], and phytochrome-mediated light signal transduction [47]. In maize, low Pi stress dramatically induced the *ZmSPX* gene (except *ZmSPX3*) [48]. Opposite to the wild type (WT), tobacco leaves with overexpression of *OsSPX*1 had lower levels of total phosphorus, reduced sucrose and free proline built up [49]. In *Arabidopsis thaliana*, anthocyanin production is negatively regulated by the Pi signaling factor *SPX4*. Under Pi supplies, *PAP1* activity can be antagonized by direct protein interactions, while Pi starvation breaks down the SPX4-PAP1 complex and binds *PAP1* to the *DFR* gene promoter to activate the biosynthesis of anthocyanins [50]. In this study, transcriptome analysis of Pi-supplied tea leaves confirmed the co-induction of *CsSPX3* and *CsPHL7*. Based on qRT-PCR, we discovered that there was a significant link between the expression of *CsSPX3*, *CsPHL7*, *CsTS1*, and *CsGS1*. In contrary to low phosphorus, high phosphorus resulted in the up-regulation of *CsSPX3* and *CsPHL7* expression levels and the inhibition of theanine accumulation. After *CsSPX3* was silenced utilizing VIGS technology, theanine content increased dramatically, but *CsSPX3* overexpression decreased theanine content. Furthermore, we explored whether the Pi state affects the binding of SPX3 and PHL7 and found that the ability of SPX3 bind to PHL7 is not significantly dependent on the Pi state. But other results showed that in the presence of high Pi, *C. sinensis* PHR1 interacted with SPX3 and co-existed with SPX3-PHL7 interaction, at which time the SPX3-PHL7 binding effect was diminished. In contrast, under low phosphorus conditions, SPX3 did not bind to PHR1. Hence, we propose SPX3 interacts with PHL7 to play an essential role in mediating Pi regulation theanine biosynthesis in tea plants.

### *CsPHL7* bound to the promoter of *CsTS1* and *CsGS1* to negatively regulate theanine biosynthesis

The regulatory mechanisms underlying the various factors-triggered theanine biosynthesis or reduction as well as the regulation of their biosynthesis-related genes are still poorly understood, even though several key genes related in theanine biosynthesis and regulation have been identified. The investigation has revealed that *CsMYB6*, which binds to the *CsTS1* promoter, positively regulates theanine production in tea roots [51], and theanine biosynthesis is negatively regulated by *CsMYB73*, which bound to *CsGS1b* and *CsGS2a* gene promoters [52]. CsAlaDC is an essential enzyme involved in producing ethylamine, a precursor for the biosynthesis of theanine. The expression of *CsAlaDC* is conjointly regulated by *CsHHO3* and *CsMYB40* in reaction to nitrogen metabolism for theanine biosynthesis [53]. *CSTS1* is primarily expressed in roots for the biosynthesis of theanine, while *CsGS1* mainly catalyzes theanine synthesis in tea leaves [33–35]. Our study strongly supported the binding of Cs*PHL7* to the consensus sequence of Cs*TS1* and *CsGS1* promoter containing multiple *cis*-elements. The analysis of gene expression also indicated that the expression pattern of *CsTS1* and *CsGS1* was closely associated with the theanine accumulation in tea plant tissues, the expression pattern of Cs*PHL7* appeared to be contrary with both Cs*TS1* and Cs*GS1* expression and the theanine contents in tea plant tissues. The results of EMSA and dual-LUC showed that CsPHL7 specifically bound to the *CsTS1* and *CsGS1* promoter and repressed its expression in tea plants. At the same time, we found that CsSPX3 interacted with CsPHL7 to additively inhibit theanine synthesis and *CsTS1、CsGS1* expression. In addition, silencing *CsPHL7* by VIGS technology led to an increase in theanine content, and overexpression of *CsPHL7* by *Agrobacterium*-mediated transient transformation system led to a decrease in theanine content. As a result, *CsPHL7* negatively regulates theanine content in tea.

### Regulation model for Pi inhibition of theanine biosynthesis mediated by *CsSPX3–CsPHL7–CsGS1/CsTS1*

The biosynthesis of theanine is affected by genetic, physiological, and environmental factors, including transcription factors, temperature, hormones, light, and nutrient availability [54]. In the regular growth conditions, Pi status is closely related to the nitrogen and carbon metabolism in tea plants. Water extracts and amino acid contents of tea leaves can be increased by applying Pi fertilizer appropriately. In this research, the production of theanine dropped and the expression levels of *CsPHL7* and *CsSPX3* increased when tea plants were cultured in high Pi conditions. On the contrary, theanine content rose when *CsSPX3* and *CsPHL7* expression levels dropped in low Pi conditions.

**Figure 8:**
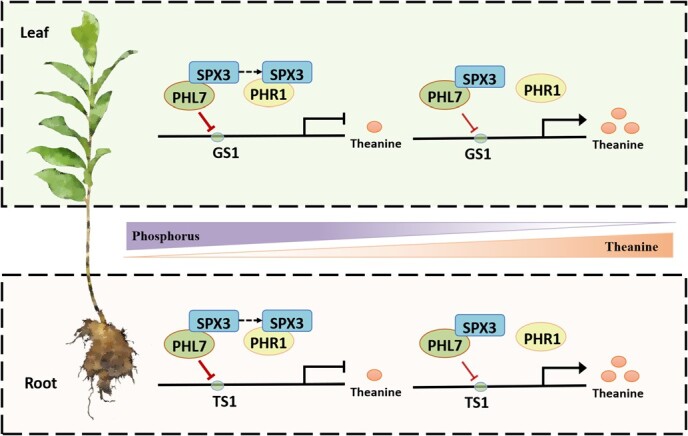
The working model for *CsSPX3–CsPHL7–CsGS1/CsTS1* mediated Pi supply inhibition of theanine biosynthesis.

In addition, theanine content significantly increased in tea plants when *CsSPX3* was silenced, and the expression levels of *CsTS1* and *CsGS1* (a downstream target gene of *CsPHL7*) was observed to be higher in *CsSPX3*-silenced plants than in control plants. Altogether, CsSPX3 could interact with CsPHL7 to participate in theanine biosynthesis, while *CsPHL7* inhibited the expression of theanine synthesis-related genes *CsTS1* and *CsGS1*, and negatively regulates the accumulation of theanine in roots and leaves in tea plants acting as a negative regulator of theanine biosynthesis. Therefore, the regulatory mechanism model of Pi signal mediated by *CsSPX3*-*CsPHL7-CsGS1/CsTS1* on theanine biosynthesis was established (Figure 8).

To summarize, our results offer guidance for molecular breeding of tea and other plant species containing theanine. For all that, the impact of the Pi element in nature on the growth, development of tea plants, and the quality of tea leaves remains a topic requiring further investigation in the future. Next, we plan to conduct in-depth studies to clarify the regulation mechanism of genes associated to theanine biosynthesis that are impacted by genetic, environmental, and other factors.

## Materials and methods

### Plant materials and treatments

The tea plant cultivars (‘Fuding Dabaicha’ and ‘Wuniuzao’) seedlings, which were naturally planted in Guizhou University, were used as experimental materials. The tea cultivars were cultivated in the Guizhou Academy of Agricultural Sciences experimental farm under regular field conditions (26 ° 11 ′ N, 106 ° 27 ′ E, average altitude of 1185 m, Guiyang). The regular hydroponic nutrient solution (the element composition is shown in Table S1 for tea plant growth contains 4 mg·L^−1^ Pi (provided by KH_2_PO_4_) were used in the hydroponic experiment. In the Pi gradient experiment, 0 mg·L^−1^ Pi, 0.5 mg·L^−1^, 1 mg·L^−1^, 2 mg·L^−1^, 8 mg·L^−1^, 16 mg·L^−1^, and 32 mg·L^−1^ Pi (Provided by KH_2_PO_4_) solutions were added respectively. The concentration of K was maintained at a constant level by the addition of K_2_SO_4_ for all treatments. Once a week, the pH of the culture medium was measured and corrected to 5.5 using 1 M NaOH [10, 11]. It was grown in a room for growth maintained at 25°C/18°C day/night with 70% relative humidity. Samples were kept at −80°C or utilized fresh, frozen in liquid nitrogen right away.

### Determination of theanine content by HPLC

The theanine extraction and detection were carried out as previously described with minor adjustments [35]. The tea tree sample roots and leaves were vaporized for 1 minute, dried to dry at 80°C, and ground into tea powder for later use. The HPLC method was used to measure the amount of theanine present in leaves and roots. The temperature of the normal phase C18 column (5 μm，250 mm×4.6 mm) was maintained at 35°C. The wavelength of 210 nm was chosen. The phase A consisted of water, and phase B consisted of 100% acetonitrile. The 10-μl extraction was put into the HPLC apparatus to measure it.

### Determination of phosphorus in plants by molybdenum-antimony anti-colorimetric method

The content of phosphorus was measured using the previously reported ascorbic acid-molybdenum antimony anti-colorimetric method [55] with slight modification. The plant samples were dried at 60°C to 70°C until they were easily ground. After cooling, they were immediately crushed and passed through a 0.25-mm sieve for use. The total phosphorus in plants was determined by molybdenum antimony colorimetric method. The colorimetric determination was performed at a spectrophotometer wavelength of 700 nm.

### Transcriptome sequencing and total RNA extraction, real-time quantitative PCR analysis

Transcriptome sequencing was conducted on triplicate samples using the Illumina NovaSeq 6000 platform by Novogene (Beijing, China). This study analyzed the FPKM values of key differentially expressed genes.

Every frozen sample was treated with the modified cetyltrimethylammonium bromide (CTAB) technique to extract total RNA [56]. The extracted RNA was reverse transcribed into cDNA and diluted 10 times for subsequent detection. The *CsACTIN* gene in tea served as an internal reference for the study. qRT-PCR was carried out using the Bio-Rad CFX Connect™ real-time quantitative PCR instrument. The gene's relative expression level was assessed using 2^−ΔCt^. Primers used are listed in Table S2.

### Subcellular localization analysis

The open reading frames (ORFs) of *CsSPX3* and *CsPHL7* were cloned from tea plant leaf cDNA libary and inserted into the GFP fusion expression PCAMBIA1300 vector to obtain fusion constructs 35S:CsSPX3-GFP and 35S:CsPHL7-GFP. The fusion construct was temporarily expressed through *Agrobacterium*-mediated transformation in tobacco epidermal cells. After injection, the cells were cultured in low light for 2 to 3 days to make glass slides. Using a laser confocal microscope, the distribution of green fluorescence signals was examined, and fluorescence pictures were taken. Primers used are listed in Table S3.

### Luciferase complementary imaging (LCI) for CsSPX3-CsPHL7 interaction

To create the CsSPX3-nLUC fusion protein expression plasmid, the open reading frame (ORF) of *CsSPX3* was cloned and ligated with the N-terminal segment of luciferase (nLUC). The ORF of *CsPHL7* was ligated with the C-terminal fragment of luciferase (cLUC) to construct CsPHL7-cLUC fusion protein expression plasmid. The plasmids were converted into *Agrobacterium tumefaciens*, and positive *Agrobacteria* transformants harboring either CsSPX3-nLUC or CsPHL7-cLUC plasmid were expressed in tobacco leaves through infiltration in different combinations. After incubation for 2 to 3 days, 1 mM d-luciferin potassium substrate solutions were applied to tobacco leaves by spraying, and the leaves were imaged under the chemiluminescence instrument Fusion FX7 (VILBER, France) after 5 to 10 minutes of darkness [57], capturing the chemofluorescence imaging system signal and take pictures. Primers used are listed in Table S3.

### Bimolecular fluorescence complementation (BiFC) for *CsSPX3-CsPHL7* interaction

The N-terminal and C-terminal segments of the YFP were fused to the ORFs of *CsSPX3* and *CsPHL7*, respectively. The resultant plasmids were converted into the *Agrobacterium tumefaciens* GV3101 strain, then selecting the positive strains and co-expressing in the *N. benthamiana* leaves [58]. After infiltration for 2 to 3 days, the YFP fluorescence in tobacco leaves was visualized using a German card Zeiss LSM900 laser confocal scanning microscope. YFP fluorescence was excited at 514 nm, and observed between 527 and 542 nm. Primers used are listed in Table S3.

### Electrophoretic mobility shift assay (EMSA)

The cDNA of *CsPHL7* was inserted into pCZN1, and the recombinant His-CsPHL7 was purified using the Ni-NTA His Bind purification kit (Novagen, Beijing, China) in accordance with the direction of manufacturer. The Lightshift TM chemiluminescence EMSA kit (Thermo Scientific, USA) was employed to perform the EMSA. Sequences of biotin-labeled probes used are listed in Table S3.

### Dual-LUC reporter assay system

Transactivation activity was analyzed in tobacco leaves by using a dual-LUC assay system [59]. The promoter fragment of *CsTS1* was inserted into the pGreenII 0800-LUC vector as a reporter gene, while the ORF of *CsPHL7* was cloned into the pGreenII 62-SK vector as an effector, which was used to transform the *Agrobacterium tumefaciens* EHA105 strain, and in the leaf cells infected with EHA105, the recombinant plasmid was then temporarily expressed through fusion. The infected leaves were sprayed with 0.2 mg·mL^-1^ D-fluorescein sodium salt after 3 to 5 days, and they were then incubated for ten minutes at 37°C. A chemiluminescence device called Fusion FX7 (VILBER, France) was used to create the fluorescence images. Using a dual-LUC reporter gene assay kit, the transcriptional activity based on the ratio of LUC/REN was determined. Primers used are listed in Table S3.

### VIGS-based gene silencing in tea plants

VIGS was employed to individually silence the *CsSPX3* and *CsPHL7* gene in the tea cultivar ‘Fuding Dabaicha’ [60]. The TRV2-*CsSPX3* and TRV2-*CsPHL7* vector was created by assembling the *CsSPX3* and *CsPHL7* fragment for VIGS into the TRV2 virus vector. Then, TRV1, TRV2, TRV2-*CsSPX3*, and TRV2-*CsPHL7* were transformed into tumefaciens GV3101 strain, respectively. Following culture and suspension, a 1:1 (v:v) ratio was used to mixed the *Agrobacterium tumefaciens* solution containing TRV1 with the *Agrobacterium tumefaciens* solution carrying TRV2, TRV2-*CsSPX3*, or TRV2-*CsPHL7*. The tea cuttings were trimmed to 20 cm using scissors, while keeping the mature leaves intact. The inoculated tea cuttings were cultivated in a greenhouse at 25°C with a 16-hour/8-hour light/dark cycle after 3 days of being kept in the dark.

### Transient overexpression of *CsSPX3* and *CsPHL7* in tea leaves

Using the previously reported transient transformation system of overexpression in tea leaves [61], with a slight modification, was employed to obtain the second leaf of ‘Wuniuzao’ with transient overexpression of *CsSPX3* and *CsPHL7*. The transformed *Agrobacterium* tumefaciens** (GV3101 chemoreceptor cells) constructed with *CsSPX3* and *CsPHL7* are cultured in liquid YEP medium. Then, it was suspended in a suspension buffer that contained 10 mM MES (pH 5.6), 10 mM MgCl_2_, and 200 μM acetylsyringone (AS) until the final OD was 1.0. Agrobacterium with pCAMBIA1300 vector carrying 35S promoter was injected into the second leaf as a control. In order to avoid the influence of different leaf growth stages on RNA expression and biochemical component content, we compared two sides of the same leaf to minimize the influence of background inconsistency on gene expression. Samples were collected, frozen in liquid nitrogen, and split into two halves for theanine content measurement and qRT-PCR after three days after injection.

## Supplementary Material

Web_Material_uhae242

## Data Availability

The data from this article can be found in the article and its supplementary tables and figures (see supplementary material). Other specific data will be available upon request from the corresponding author.
