# Supplementary material for: CsSPX3-CsPHL7-CsGS1/CsTS1 module mediated Pi-regulated negatively theanine biosynthesis in tea (Camellia sinensis)
Source: Hortic Res. 2024 Aug 30;11(11):uhae242. doi: 10.1093/hr/uhae242 (PMC11554760; doi:10.1093/hr/uhae242)
Supplement: Web_Material_uhae242 [file web_material_uhae242.zip › Revised Supplementary File.pdf]

CsSPX3-CsPHL7-CsGS1/CsTS1 module mediated Pi-regulated negatively theanine biosynthesis in tea (*Camellia sinensis*)

Zhouzhuoer Chen[1], Zhixun Yu[1], TingTing Liu[1], Xinzhuan Yao[2], Shiyu Zhang[1], Yilan Hu[1], Mingyuan Luo[1], Yue Wan[3], Litang Lu[1,*]

1 The Key Laboratory of Plant Resources Conservation and Germplasm Innovation in Mountainous Region (Ministry of Education), College of Life Science, Guizhou University, Guiyang 550025, China.

2 College of Tea Science, Institute of Plant Health & Medicine, Guizhou University, Guiyang, 550025, China.

3 Huaneng Clean Energy Research Institute, Beijing 102209, China

* Corresponding author: ltlv@gzu.edu.cn; Tel.: +86-13639031105

E-mail addresses: gs.zzmchen21@gzu.edu.cn (Z. Chen); ls.zxyu22@gzu.edu.cn (Z. Yu); ls.ttliu22@gzu.edu.cn (T. Liu); xzyao@gzu.edu.cn (X. Yao); gs.hurj22@gzu.edu.cn (S. Zhang); gs.huyl21@gzu.edu.cn (Y. Hu); gs.myluo21@gzu.edu.cn (M. Luo); y_wan@qny.chng.com.cn(Y. Wan); ltlv@gzu.edu.cn (L. Lu *)

The following Supporting Information is available for this article:

Figure S1. The changes of catechin content and caffeine content in tea plants treated with different concentrations of phosphorus.

Figure S2. Gene co-expression modules associated with theanine content under Pi-supply conditions based on WGCNA.

Figure S3. Phylogenetic relationship of SPX/PHO proteins in various species.

Figure S4. Subcellular localization of CsSPX3 and CsPHL7 protein under low Pi and high Pi conditions.

Figure S5. Phylogenetic analysis of the evolutionary relationship of *CsPHRs*.

Figure S6. The protein of CsPHL7-His was successfully expressed and extracted.

Figure S7. CsPHL7 binds to *CsGS1* Promoter.

Figure S8. Pi-supply inhibited the accumulation of theanine in *CsSPX3* and *CsPHL7* silenced plants.

Figure S9. Transient over-expression injection method in tea plant.

Figure S10. Pi-supplying-inhibited theanine was significantly lower in *CsSPX3* and *CsPHL7* overexpressed plants.

Table S1. Composition of standard culture solution.

Table S2. Primers for qRT-PCR.

Table S3. List of primers used in this study.

Table S4. Expression prifiles of genes for Pi treatment.

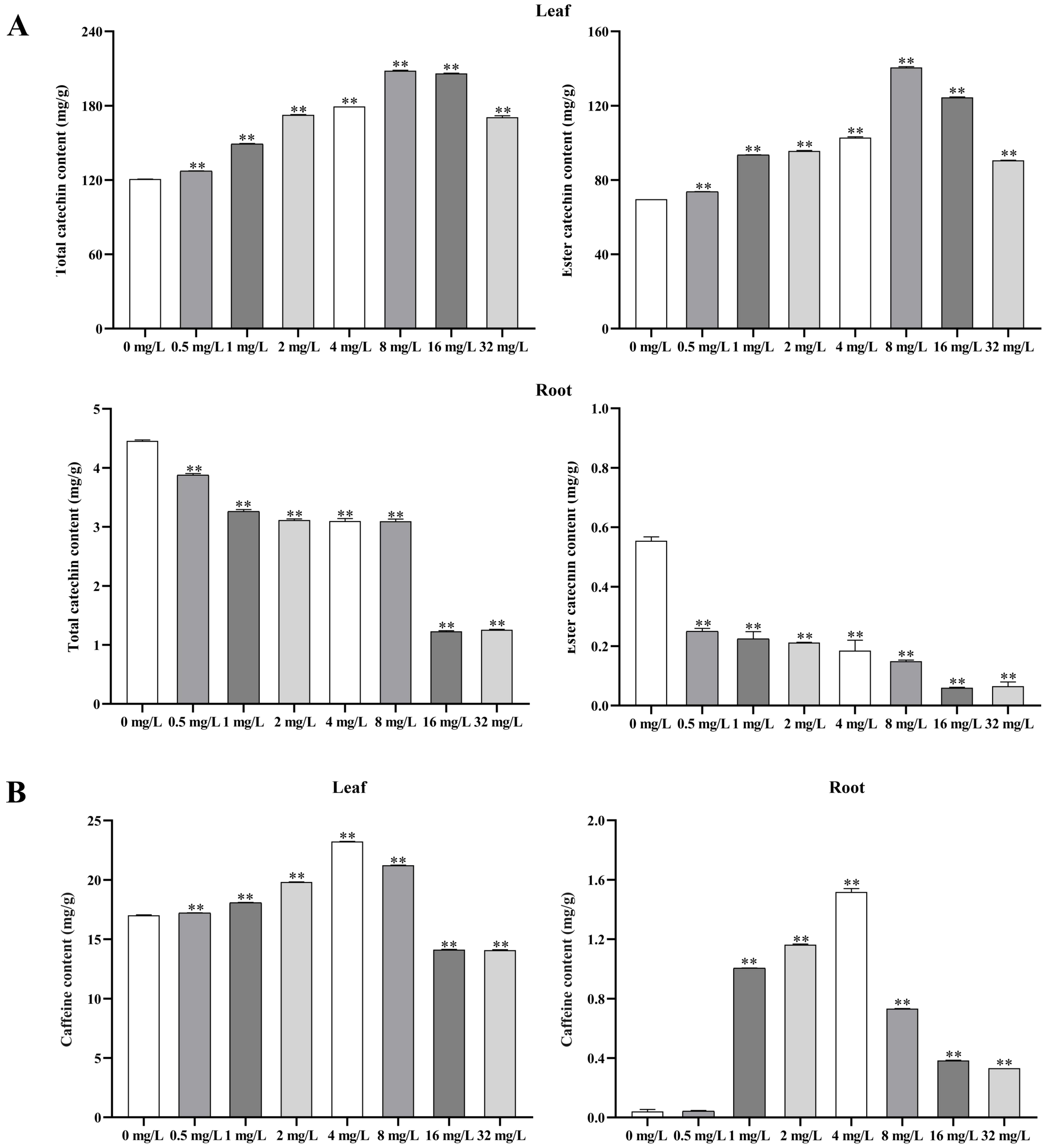

Figure S1. The changes of catechin content and caffeine content in tea plants treated with different concentrations of phosphorus. (A) The total catechins and ester catechins in tea leaves and roots on the 20th day of different phosphorus concentration treatment. (**B)** The caffeine content in tea leaves and roots on the 20th day of different phosphorus concentration treatment. The error bars represent the mean ± SE of the three independent experiments. * indicate significant differences at $p < 0.05$ by Duncan' s multiple range tests. SE, standard error.

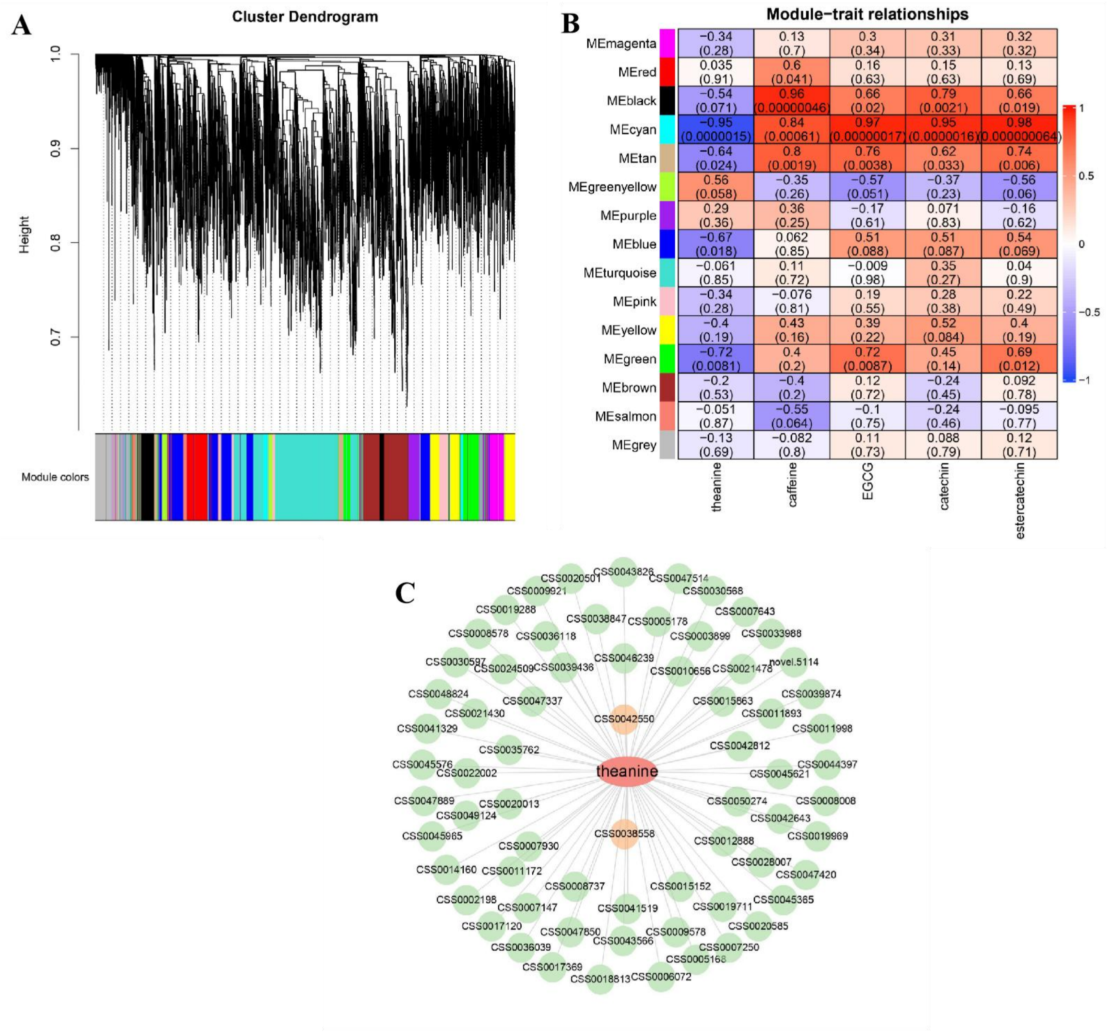

Figure S2. Gene co-expression modules associated with theanine content under Pi-supply conditions based on WGCNA. (A) Clustering dendrograms of genes. **(B)** Heat map of correlations between modules and traits. **(C)** The regulatory network diagram of the core genes in the cyan modules.

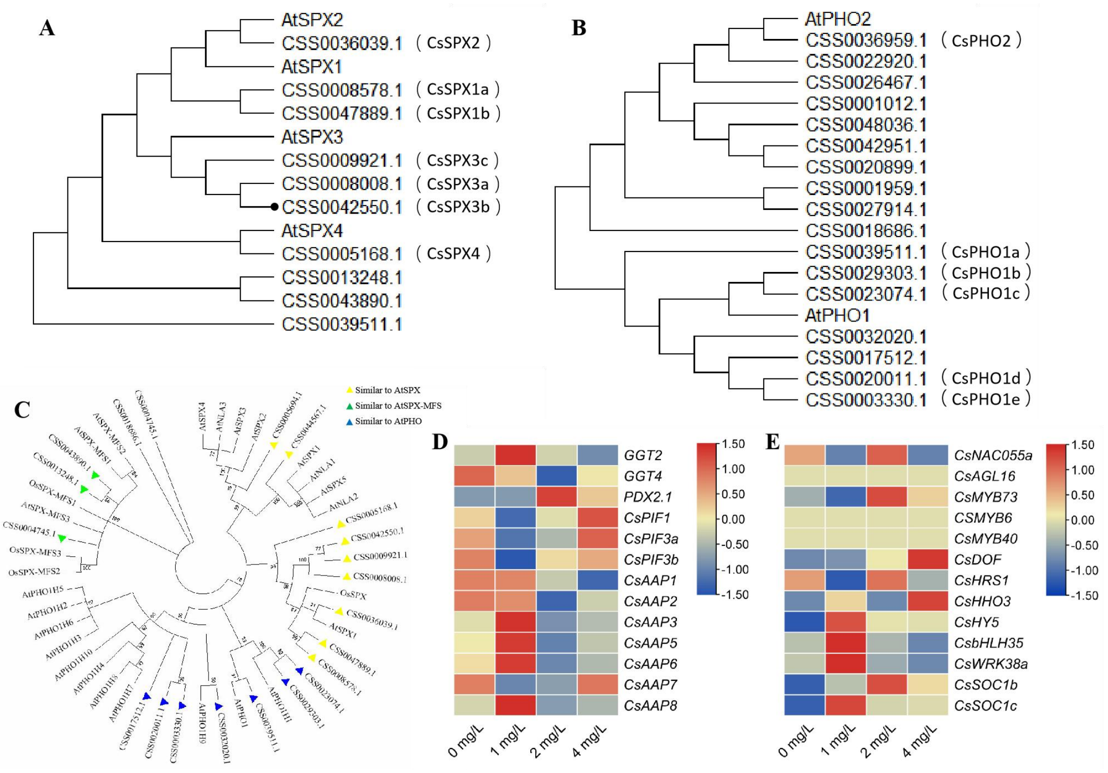

Figure S3. Phylogenetic relationship of SPX/PHO proteins in various species. (A) Phylogenetic analysis of *CsSPX* and Arabidopsis SPX proteins. **(B)** Phylogenetic analysis of *CsPHO* and Arabidopsis PHO proteins. **(C)** Phylogenetic tree of SPX/PHO proteins in *Camellia sinensis*, *Arabidopsis* and rice. Candidate genes in tea plants were highlighted by black circle. The numbers at the nodes indicate the bootstrap value with 1000 replicates. RNA-seq transcriptome heat map of genes related to **(D)** theanine degradation and **(E)** inhibition of theanine biosynthesis.

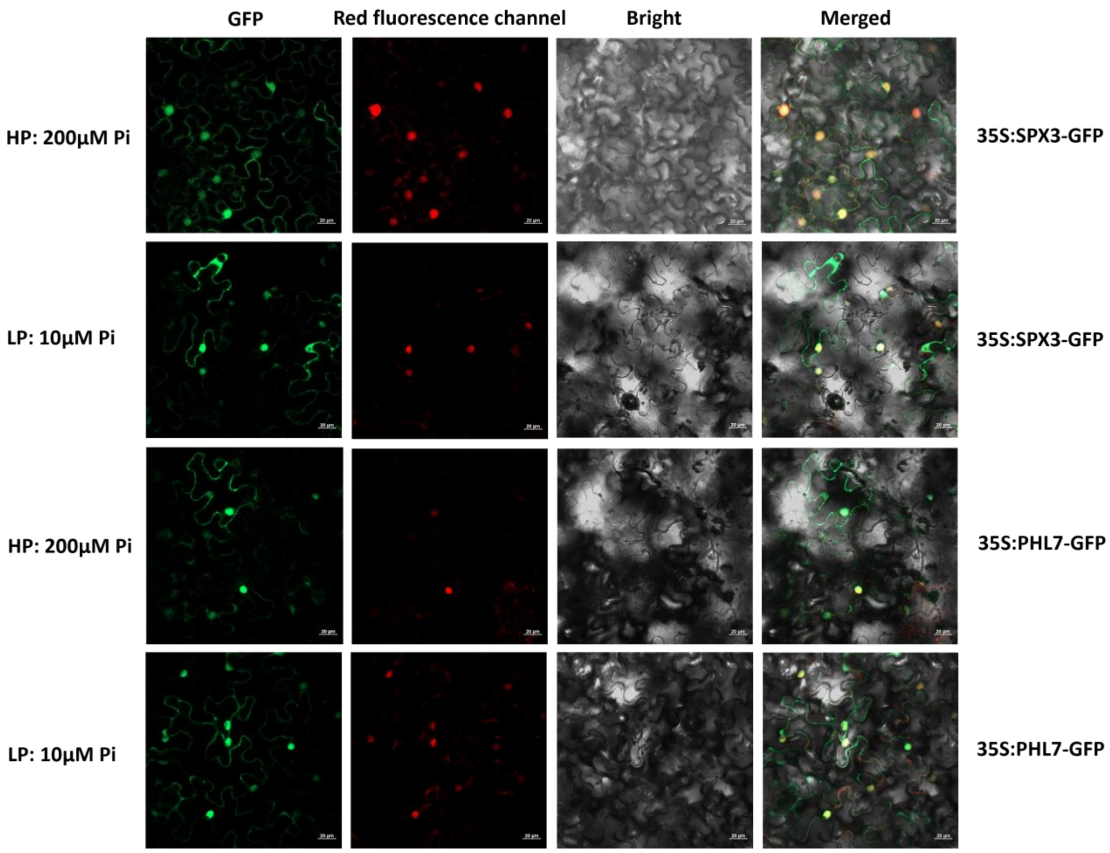

Figure S4. Subcellular localization of CsSPX3 and CsPHL7 protein under low Pi and high Pi conditions. Confocal images of 35S:SPX3-GFP and 35S:PHL7-GFP in N. benthamiana leaves localizing to the nucleus and cell membrane under low Pi （LP;5 μM Pi）and high Pi（HP;200 μM Pi）conditions. Scale bar = 20 μm.

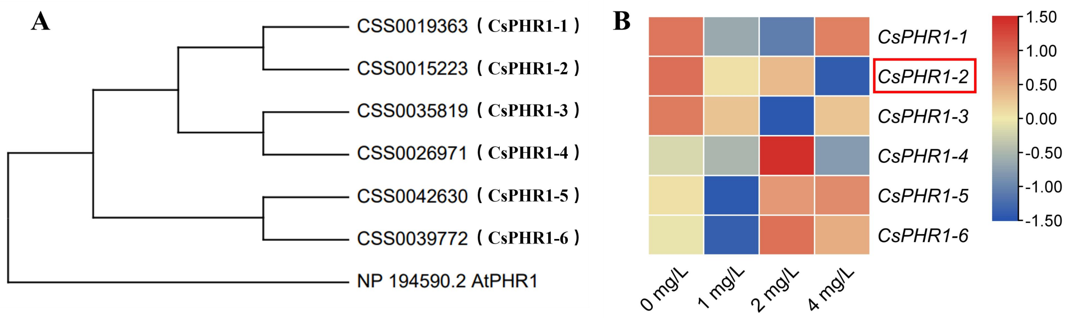

Figure S5. Phylogenetic analysis of the evolutionary relationship of CsPHRs.

(A) Phylogenetic tree of PHR family proteins from Camellia sinensis, Arabidopsis generated. Identifiers for the PHR genes are listed in. **(B)** RNA-seq transcriptome profiles of *CsPHRs* genes under different phosphorus concentrations.

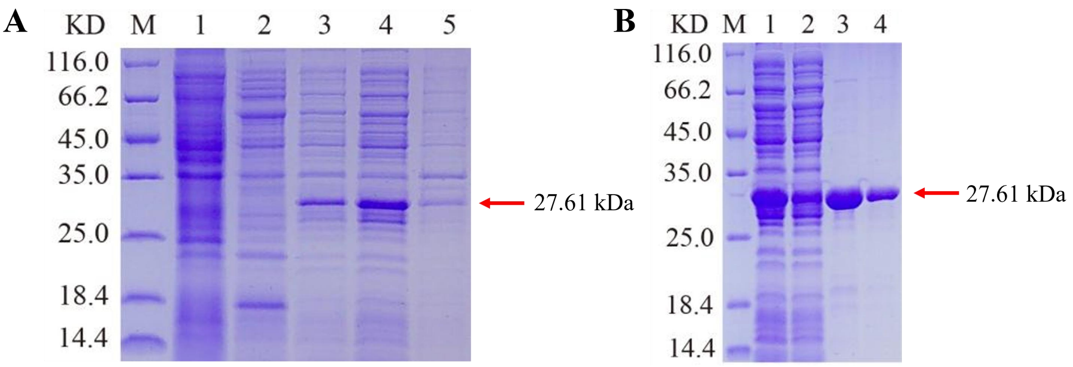

Figure S6. The protein of CsPHL7-His was successfully expressed and extracted. **(A)** SDS polyacrylamidegel electrophoresis detected the protein of CsPHL7-His were successfully expressed. M: Quality standard for protein molecules; 1: pCZN1 induction (no-load); 2: Not induced; 3: After induction; 4: Induced supernatant after crushing; 5: Precipitation after induced crushing. **(B)** SDS polyacrylamidegel electrophoresis detected the protein of CsPHL7-His were successfully purified. M: Quality standards for protein molecules; 1: Crushing the post-processing sample; 2: Outflow; 3-4: Elution. Red arrow denotes target protein bands with corresponding molecular weight.

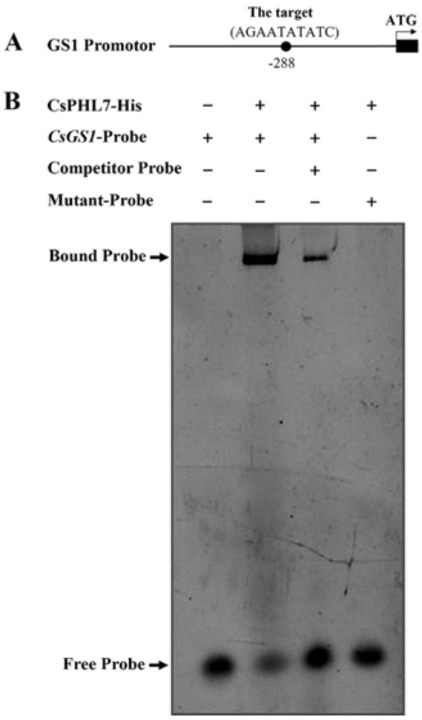

Figure S7. CsPHL7 binds to *CsGS1* Promoter. (A) Position of the CsPHL7 predicted binding targets on the CsGS1 promoter. The solid circles stand for binding site, and the arrow indicates transcription start site. **(B)** Recombinant His-PHL7 was purified from E. coli. The promoter fragment containing the binding motifs of the GS1 promoter was labeled by biotin. Competition for the protein-DNA binding was performed using 100X unlabeled wild-type probes (Competitor). The target probes TATGAAAGAATATATCATCCATACT (5′ to 3′) were replaced with TATGAAAAAAAAAAAAATCCATACT (Mutant). All the probe sequences are listed in Supporting Information Table S3.

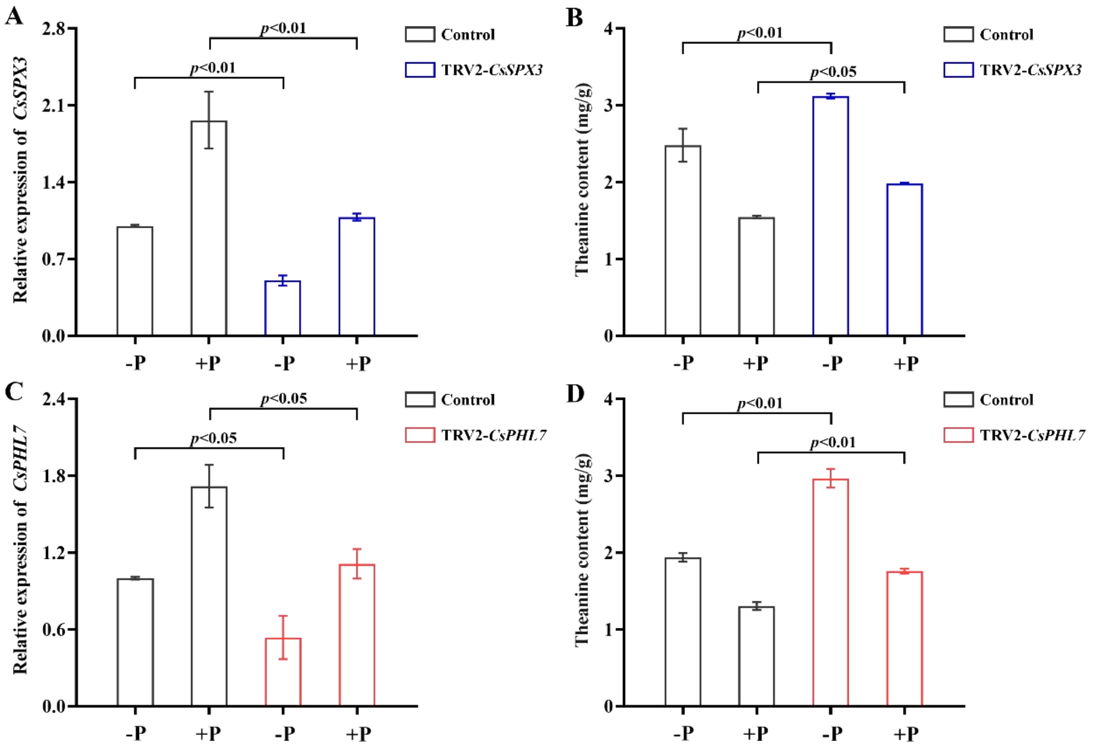

Figure S8. Pi-supply inhibited the accumulation of theanine in *CsSPX3* and *CsPHL7* silenced plants. (A) The relative expression of control and *CsSPX3* silenced plants under the -P and +P conditions. **(B)** The theanine content of control and *CsSPX3* silenced plants under the -P and +P conditions. **(C)** The relative expression of control and *CsPHL7* silenced plants under the -P and +P conditions. **(D)** The theanine content of control and *CsPHL7* silenced plants under the -P and +P conditions.

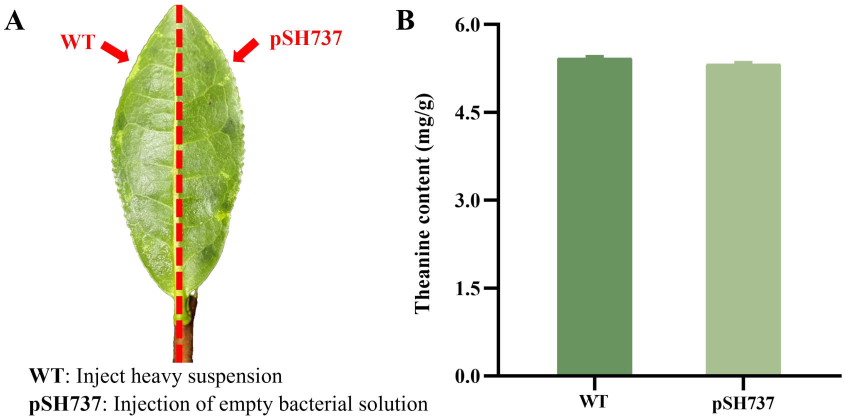

Figure S9. Transient over-expression injection method in tea plant. (A) Three sets of leaves were injected into the same leaf position (left: WT, right: pSH737). **(B)** Theanine content in the left and right parts of the single leaf. To show that no-load (pSH737) will not affect the material contained in the plant. And the subsequent experimental groups were compared with the empty group. The error bars represent the mean ± SE of the three independent experiments. * indicate significant differences at $p < 0.05$ by Duncan' s multiple range tests. SE, standard error.

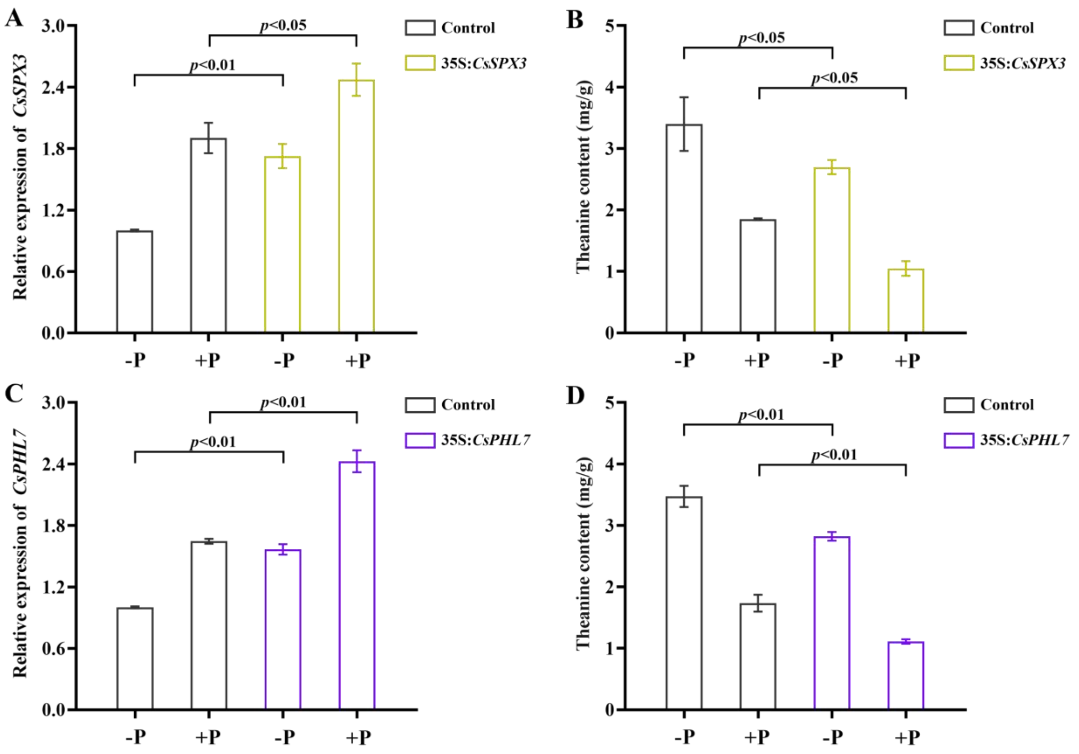

Figure S10. Pi-supplying-inhibited theanine was significantly lower in *CsSPX3* and *CsPHL7* overexpressed plants. (A) The relative expression of control and *CsSPX3* overexpressed plants under the -P and +P conditions. **(B)** The theanine content of control and *CsSPX3* overexpressed plants under the -P and +P conditions. **(C)** The relative expression of control and *CsPHL7* overexpressed plants under the -P and +P conditions. **(D)** The theanine content of control and *CsPHL7* overexpressed plants under the -P and +P conditions.

Table S1. Composition of standard culture solution.

Element	Compound	Conoentration ($mg \cdot L^{-1}$)
N	$(NH_4)_2SO_4$	30
P	KH_2PO_4	4
K	K_2SO_4, KH_2PO_4	40
Ca	$CaCl_2 \cdot 4H_2O$	20
Mg	$MgSO_4 \cdot 7H_2O$	25
Al	$Al_2(SO_4)_3 \cdot 18H_2O$	21
Fe	Fe-EDTA	0.35
B	H_3BO_3	0.1
Mn	$MnSO_4 \cdot 4H_2O$	1
Zn	$ZnSO_4 \cdot 7H_2O$	0.1
Cu	$CuSO_4 \cdot 5H_2O$	0.03
Mo	$Na_2MoO_4 \cdot 2H_2O$	0.05

Table S2. Primers for qRT-PCR.

Primer name	Primer sequence（5′ to 3′）
SPX3-qPCR-F	CGGAGAAAGAGAGGCGATTATG
SPX3-qPCR-R	AAGTTGAGAGGCGGTAGAGA
PHL7-qPCR-F	CGCTTCGTAGACGCTATTACTC
PHL7-qPCR-R	GGTTCACCCATCACTCGTAAA
TS1-qPCR-F	GACAAGCTCCTGGTGAAGATAG
TS1-qPCR-R	CCATTCCAATCACCCTCGATAG
TS2-qPCR-F	AGGCATTGCGTAGAGTTTCA
TS2-qPCR-R	CAGAACAGTAGGGCGTCATATC
GS1-qPCR-F	CCTCAGAAGCAAAGCAAGGACT
GS1-qPCR-R	AACATCAGGGTGGCTGAAAATC
GS2-qPCR-F	GTGGATGGCTCGTTACATTCT
GS2-qPCR-R	CCATTCCAGTCACCCTCAATAG

Actin-qPCR-F	CAGACCGTATGAGCAAGGAAAT
Actin-qPCR-R	GTGCTTAGGGATGCAAGGATAG

Table S3. List of primers used in this study.

Probe Name	Primer sequence（5′ to 3′）	purpose
GFP- SPX3-F	AACACGGGGGACGAGCTCGGTACCATGAAATT TGGGAAGAGATTGA	Subcellular localization
GFP- SPX3-R	CTCGCCCTTGCTCACCATGTCGACGGGAATTGG TATGGGTGAAT	Subcellular localization
GFP- PHL7-F	TTGGAGAGAACACGGGGGACGAGCTCATGTAT CACGCCAAGAA	Subcellular localization
GFP- PHL7-R	CCTTGCTCACCATGTCGACTCTAGAGGATCCGT CCTCTTCTGGTGGGAC	Subcellular localization
1300nLUC- SPX3-F	GAGCTCGGTACCCGGGATCCTCTAGAATGAAATTTGG GAAGAGATTGA	LCI
1300nLUC- SPX3-R	CGGGACGCGTACGAGATCTGGTCGACGGGAATTGGTA TGGGTGAAT	LCI
1300cLUC- PHL7-F	GGGCGGTACCCGGGATCCTCTAGAGATGTATCACGCC AAGAAATTTTC	LCI
1300cLUC- PHL7-R	ACGAACGAAAGCTCTGCAGGTCGACCTAGTCCTCTTC TGGTGGGAC	LCI
1300cLUC- PHR1-F	AGGACAGCCCAAGCTGAGCTCCACCGCGGTGGCGGC CGCATGGAGAGAGAACTGAATAC	LCI
1300cLUC- PHR1-R	CCCCCTCGAGGTCGACGGTATCGATAAGCTTTCATTCA GTATTAGCAAAGAAG	LCI
SPX3-nYFP-F	CACGGGGGACGAGCTCGGTACCATGAAATTTGGGAAG AGATTGAAGC	BiFC
SPX3-nYFP-R	TCAACTTTTGCTCCATGTCGACGGGAATTGGTATGGGT GAATTGAG	BiFC

PHL7-cYFP-F	CACGGGGGACGAGCTCGGTACCATGTATCACGCCAAG AAATTTTC	BiFC
PHL7-cYFP-R	TCGTATGGGTACATGTCGACGTCCTCTTCTGGTGGGAC	BiFC
TS1-Probe-1-F	AATTAACCTTATCATATTCATAACCTAAG	EMSA
TS1-Probe-1-R	CTTAGGTTATGAATATGATAAGGTTAATT	EMSA
TS1-Probe-2-F	AATCATGACATTGATATTCCCAAAAAATG	EMSA
TS1-Probe-2-R	CATTTTTTGGGAATATCAATGTCATGATT	EMSA
TS1-Probe-3-F	CTACCAAAAAAAGTTAGATTCCCCAAAAG	EMSA
TS1-Probe-3-R	CTTTTGGGGAATCTAACTTTTTTTGGTAG	EMSA
TS1-Probe-4-F	TAACTCTAACTCATTATTCTTGAAACCTC	EMSA
TS1-Probe-4-R	GAGGTTTCAAGAATAATGAGTTAGAGTTA	EMSA
GS1-Probe-F	Bio-TATGAAAGAATATATCATCCATACT	EMSA
GS1-Probe-R	Bio-AGTATGGATGATATATTCTTTCATA	EMSA
GS1-Competing probe-F	TATGAAAGAATATATCATCCATACT	EMSA
GS1-Competing probe-R	AGTATGGATGATATATTCTTTCATA	EMSA
GS1-Mutated Probe-F	TATGAAAAAAAAAAAAATCCATACT	EMSA
GS1-Mutated Probe-R	AGTATGGATTTTTTTTTTTTTCATA	EMSA
His-PHL7-F	TCACAAAGTGCATCATCATCATCATCATATGTAT CACGCCAAGAAATTTTCAACTG	EMSA
His-PHL7-R	TGTGCTTTTAAGCAGAGATTACCTATCTAGATTA GTCCTCTTCTGGTGGGACAAAGT	EMSA
pGreen-TS1-F	GAGGTCGACGGTATCGATAAGCTTATCTAATAG GTAAGGTCTTGTTTGGGGATA	Dual-LUC

pGreen-TS1-R	ATTCGATCTCCACCGCGGTGGCGGCCGCGGCTC TCTCTGTCTCTCTCGCTCTGCTT	Dual-LUC
pGreen-GS1-F	ACTCACTATAGGGCGAATTGGGTACCCGAAACT CATTTTTTATGATTGAATT	Dual-LUC
pGreen-GS1-R	GCGGCCGCTCTAGAACTAGTGGATCCAGTGAC GATCGATCAAAAACACCCACTCCAAA	Dual-LUC
pGreen-SPX3-F	AGGACAGCCCAAGCTGAGCTCCACCGCGGTGG CGGCCGCATGAAATTTGGGAAGAGATTG	Dual-LUC
pGreen-SPX3-R	CCCCCTCGAGGTCGACGGTATCGATAAGCTTTT AGGGAATTGGTATGGGTGAATTG	Dual-LUC
pGreen-PHL7-F	TCCACCGCGGTGGCGGCCGCTCTAGAATGTATC ACGCCAAGAAATTTTCAAC	Dual-LUC
pGreen-PHL7-R	GCGTACCGAATTGGTACCGGGCCCCCCCTCGA GCTAGTCCTCTTCTGGTGGGACAA	Dual-LUC
